# Using Hydrophilic Ionic Liquid, [bmim]BF_4_ – Ethylene Glycol System as a Novel Media for the Rapid Synthesis of Copper Nanoparticles

**DOI:** 10.1371/journal.pone.0029131

**Published:** 2012-01-06

**Authors:** Manika Dewan, Ajeet Kumar, Amit Saxena, Arnab De, Subho Mozumdar

**Affiliations:** 1 Department of Chemistry, University of Delhi, Delhi, India; 2 Department of Microbiology and Immunology, Columbia University Medical Centre, New York, New York, United States of America; RMIT University, Australia

## Abstract

In this work, we present a novel method for the synthesis of copper nanoparticles. We utilize the charge compensatory effect of ionic liquid [bmim]BF_4_ in conjunction with ethylene glycol for providing electro-steric stabilization to copper nanoparticles prepared from copper sulphate using hydrazine hydrate as a reducing agent. The formed copper nanoparticles showed extended stability over a period of one year. Copper nanoparticles thus prepared were characterized by powder X-ray diffraction measurements (pXRD), transmission electron microscopy (TEM) and quasi elastic light scattering (QELS) techniques. Powder X-ray diffraction (pXRD) analysis revealed relevant Bragg's reflection for crystal structure of copper. Powder X-ray diffraction plots also revealed no oxidized material of copper nanoparticles. TEM showed nearly uniform distribution of the particles in methanol and confirmed by QELS. Typical applications of copper nanoparticles include uses in conductive films, lubrication and nanofluids. Currently efforts are under way in our laboratory for using these nanoparticles as catalysts for a variety of organic reactions.

## Introduction

Metal nanoparticles play a significant role in diverse fields such as optical, electronic, catalysis, medical, magnetic, information storage and surface enhanced raman scattering (SERS) [1]. Copper is one of the classic metal nanoparticle systems that has gained considerable attention in the past two decades due to its unusual properties, leading to potential applications in many fields. Non-agglomerated, spherical, uniform copper nanoparticles have been employed extensively for conductive films, lubrication, nanofluids, catalysis, etc. A number of methods such as microemulsion, reverse micelles, reduction of aqueous copper salts, gamma irradiation, UV light irradiation, protecting shells by Pileni et al, electrolytic techniques by controlling electrode potential and the polyol process have been developed for the preparation of copper metal nanoparticles [2]. Chen and Sommers have described a one phase system for the synthesis of copper nanoparticles with an alkanethiolate as a protecting monolayer [3]. Sonochemical method and thermal decomposition method have also been reported [4], [5]. However, the copper nanoparticles resulting from these methods had shortcomings, like limited size, monodispersity and are susceptible to oxidation. So there is a crucial need to develop a method to synthesize copper nanoparticles with prolonged stability and in this respect ionic liquids have developed as a source of potential rescue.

The use of Ionic Liquids (ILs) in science and its technological applications is not new as they have already emerged as a green alternative to the conventional and environmentally detrimental volatile solvents. They have attracted a great deal of attention due to their high thermal stability, good conductivity, non volatility, non flammability, suitable polarity, wide electrochemical window and recyclability [6]. Most importantly physical and chemical properties of ILs can be exploited by altering their cation, anion and attached substituents [7], [8]. Hence ILs have been used extensively and have great potentiality in the upcoming applications in sensors [9], material synthesis [10]–[12], separation and extraction [13], asymmetric synthesis [14], nuclear fuel cycle processing [15], liquid thermal storage media and heat transfer fluids [16], lubricants [17], etc.

Our study is focused towards the ionic liquids based on imidazolium cations as they are especially favorable for green industrial applications [18]. Their most important property being, although they are liquid at room temperature they are considered safer and more environmentally benign as unlike organic solvents they do not evaporate [19]. Imidazolium ILs provide an excellent medium for the formation and stabilization of transition metal nanoparticles. It is bcause of the negligible vapor pressure, the size and shape of metal nanoparticles can be investigated in situ by TEM [20], [21]. Moreover particles synthesized in organic solvents are usually immiscible in water and this also limits their applicability. Many applications require for nanoparticles to be dispersed and stable in water. However, water based synthesis of nanoparticles is fraught with many problems such as ionic interactions, low reactant concentration, and difficulty in removing the stabilizers [22]. Ionic liquids present an encompassing solution as both the cation and anion of an ionic liquid can potentially serve as charge compensating groups in the synthesis procedure. When an ionic liquid is used as a reaction media the solute is solvated by ions only. Thus, the reaction can proceed in an environment totally different from that when water or ordinary organic solvents are used. As a result, high selectivity is possible [23], [24]. This study provides an alternative to synthesizing nanomaterial with minimal energy consumption and high yield. Such metal nanoparticles has been previously demonstrated to efficiently catalyze a variety of organic reactions [25]–[29]. We report herein, well dispersed, size controlled synthesis of copper nanoparticles in ionic liquid - ethylene glycol system with hydrazine hydrate as reducing agent without the aid of any heating or microwave irradiation (Supporting Information S1).

## Materials and Methods

### 1. Materials

Copper sulphate pentahydrate (CuSO_4_.5H_2_O), ethylene glycol, hydrazine hydrate (NH_2_NH_2_.2H_2_O), sodium tetrafluoroborate and 1-butyl-3-methylimidazoliumbromide were all of analytical grade and used as such. All the aldehydes and solvents were purchased from spectrochem Pvt. Ltd. Mumbai (India) and were used without any additional purification. All reactions were monitored by thin layer chromatography (TLC) on gel F254 plates. ^1^H-NMR and ^13^ C-NMR spectra were recorded in CDCl_3_ and DMSO-d_6_ on a Jeol JNN ECX- 400P spectrometer; Melting points were recorded on SECOR Laboratories instruments melting point instruments. The infrared spectra were recorded using a model Perkin Elmer spectrum BX2 FT-IR system. Spectra were recorded with Spectrum V 5.3.1 software in the range 4000–400 cm^−1^. The KBr pellet technique was adopted for recording the spectra.

### 2. Synthesis of ionic liquid [bmim]BF_4_

**Figure 1:**
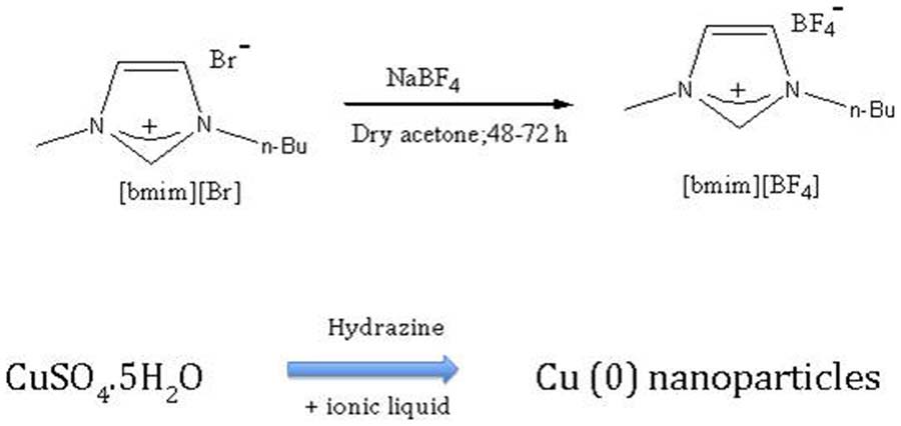
Synthesis of ionic liquid [bmim]BF_4_ and preparation of copper nanoparticles.

Sodium tetrafluoroborate and 1-butyl-3-methylimidazoliumbromide in equimolar quantities were stirred in dry acetone under anhydrous conditions for 48–72 h. The mixture was filtered off to remove unreacted sodium tetrafluoroborate and the filtrate was further treated with dichloromethane to remove sodium bromide and again the filtrate obtained was again treated with dichloromethane to check for any further precipitation. The solvents were removed under reduced pressure and the resulting colorless ionic liquid was dried in rotavapor at 70°C for 2 h to remove water. The product 1-butyl-3-methylimidazoliumtetrafuoroborate was characterized by ^1^H NMR studies. (Figure 1)

### 3. Preparation of copper nanoparticles in IL-Ethylene glycol media using hydrazine hydrate as reducing agent

In a typical experiment 5 ml ethylene glycol was used as the solvent and 100 µl of ionic liquid was added to it to give a final concentration of 1 M. This was followed by addition of 0.1 M CuSO_4_.5H_2_O (100 µl) and the reaction was allowed to stir on a magnetic stirrer under nitrogen atmosphere. After five minutes 3.0 M hydrazine hydrate (100 µl) was added as the reducing agent drop wise continued over a period of ten minutes and the system was stirred for another 30 minutes. (Figure 1). The mixture attained a uniform brown color without any aggregation. An aliquot amount of ‘ionic liquid-ethylene glycol’ protected copper nanoparticles were taken out and particle size distribution measurements were done using particle size analyzer (QELS, Photocor-FC, model-1135 P).

The particle synthesis procedure was repeated for a bulk set of 100 ml. Particles from the reaction mixture were centrifuged and washed with ethanol. The process of centrifuge and washing the particle was repeated thrice to afford powdered copper nanoparticles. Transmission electron microscope (TEM, FEI Technai 300 kV fitted with EDAX) was used to image size and morphology of the powder. X-ray diffraction patterns of the powders were recorded using diffractometer (Philips Analytica PW 1830 X-ray equipped with a 2θ compensting slits).

## Results and Discussion

We report herein a method to synthesize stabilized copper nanoparticles using hydrazine hydrate as a reducing agent in the presence of ethylene glycol and [bmim]BF_4_. We discover that ionic liquid in conjunction with ethylene glycol play a vital role in the stabilization of copper nanoparticles.

Ionic liquid [bmim]BF_4_, serves as an excellent media for dispersing copper nanoparticles, controlling their size and preventing their aerial oxidation; however agglomeration could not be avoided in the absence of ethylene glycol. Nanoparticles could also be synthesized in ethylene glycol alone. But the synthesized nanoparticles are not stable for extended periods of time. The probable reason is that the synthesized nanoparticles are oxidized. We intend to use the synthesized nanoparticles for catalysts over multiple cycles and store them. Thus our aim is to make nanoparticles that are stable for extended periods of time. Thus, we find that both ethylene glycol and ionic liquid [bmim]BF_4_ is required for synthesizing stable, monodispersed nanoparticles. Using ethylene glycol alone in the absence of [bmim]BF_4_ gives unstable nanoparticles; whereas using [bmim]BF_4_ alone in the absence of ethylene glycol leads to agglomeration of nanoparticles. Furthermore, we determine that using hydrazine hydrate as a reducing agent in the presence of ethylene glycol and hydrophilic ionic liquid [bmim]BF_4_ could serve effectively to increase the reaction and nucleation rates without employing any source of heating or microwave irradiation.

Since the ionic liquid [bmim]BF_4_ is hydrophilic, there was the possibility of it absorbing moisture thereby rendering the nanoparticles unusable over long periods of time. Our experiments showed that the nanoparticles were stabilised for at least one year. Ionic liquid [bmim][BF_4_] has been demonstrated previously to be a moisture and thermally stable compound [30]. and has been used it in the solvothermal synthesis of the coordination polymer [Cu(I)(bpp)]BF_4_. Our results are in agreement with the previous work; the added stability could also be attributed to the use of an organic solvent like ethylene glycol.

**Figure 2:**
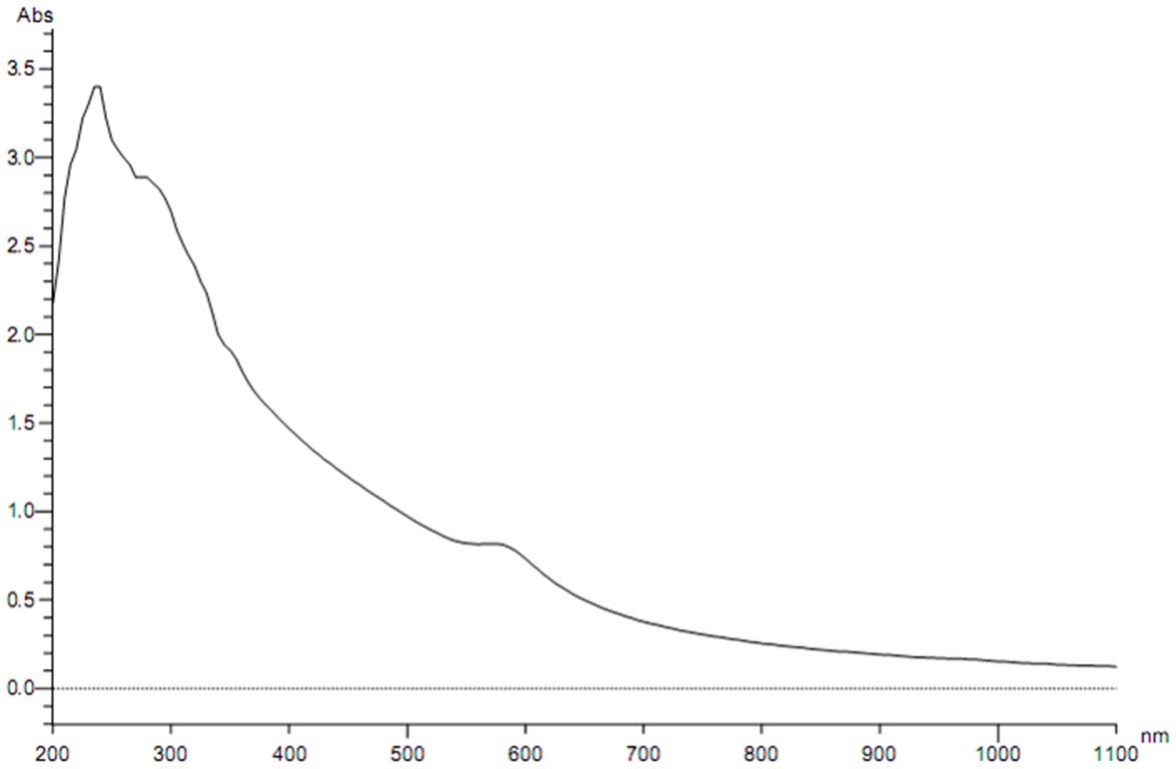
UV-Visible spectra of Copper nanoparticles.

Color changes during course of reduction indicate complex formation between copper sulphate and hydrazine hydrate. As the hydrazine hydrate solution is introduced drop wise, the blue color of copper sulphate solution turns darker and gradually turns intense yellow and eventually brown during the course of the reaction, indicating formation of highly dispersed metallic copper. UV-Vis spectra as shown in Fig. 2. It indicates formation of protected nanoparticles with λ_max_ near 580 nm.

**Figure 3:**
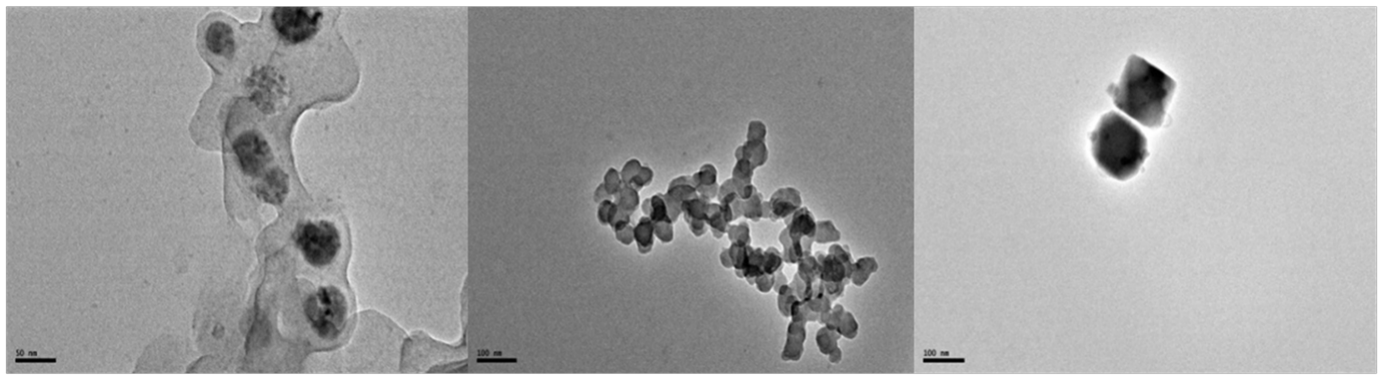
TEM images of Copper nanoparticles.

**Figure 4:**
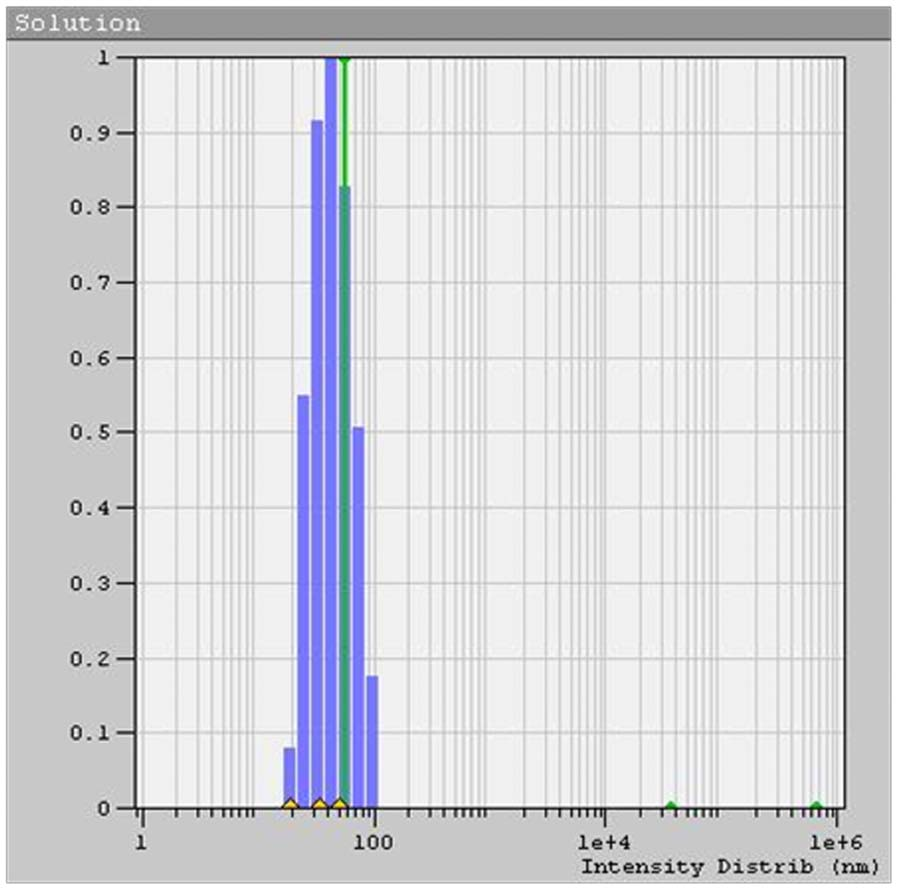
Dynamic Light Scattering data of copper nanoparticles.

**Figure 5:**
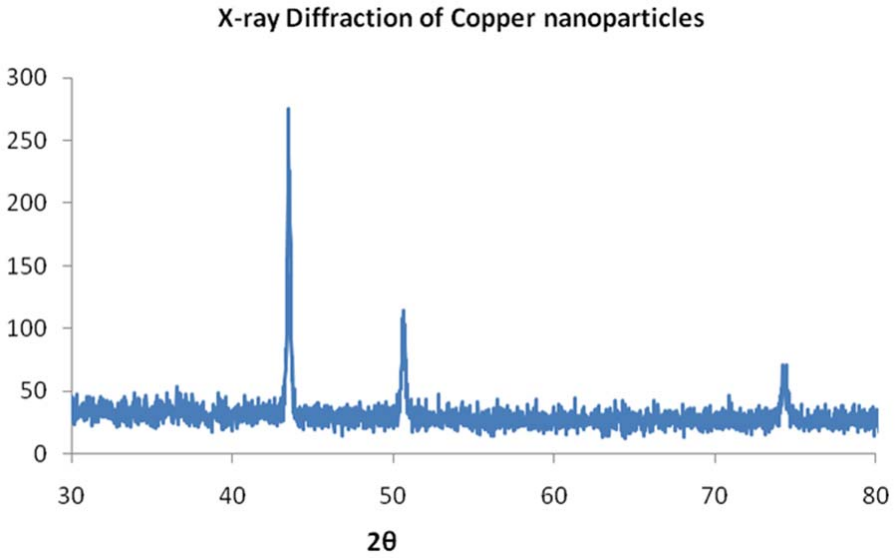
XRD plot of Copper nanoparticles.

Copper nanoparticles separated from the suspension were analyzed for their size distribution and shape by DLS and TEM. Alongside average sizes were calculated from XRD peak data. TEM images in Fig. 3 illustrates the formation of spherical, ‘ionic liquid-ethylene glycol’ protected copper nanoparticles with little agglomeration. Fig. 4 shows size distribution from the DLS data, revealing mean size of 40±3 nm in diameter with polydispersity index of 0.203. XRD patterns of the copper nanoparticles prepared from this procedure as plotted in Fig. 4, displays 2θ values from 20° to 90°. XRD pattern reflections correspond to that of pure copper (Fig. 5), shows three characteristic peaks at respectively corresponding to indices; (111), (200) and (220). These peaks accurately resemble impurity less and oxide less FCC copper phase.

### Discussion

Use of ionic liquid-ethylene glycol media for the synthesis of copper nanoparticles stabilised over a period of more than one year has been demonstrated. In the first step, there is an ion-exchange reaction where [bmim][Br] reacts with NaBF_4_ to give ionic liquid [bmim][BF_4_] and NaBr. The subsequent synthesis of Cu nanoparticles involves reduction of CuSO_4_.5H2O by hydrazine in the presence of the ionic liquid (Scheme 1). A simple, convenient and significant method for the reductive synthesis of copper nanoparticles in ionic liquid has thus been uncovered here.

## Supporting Information

Supporting Information S1 Graphical Abstract.
(DOC)
