# Supplementary material for: Using Hydrophilic Ionic Liquid, [bmim]BF4 – Ethylene Glycol System as a Novel Media for the Rapid Synthesis of Copper Nanoparticles
Source: PLoS One. 2012 Jan 6;7(1):e29131. doi: 10.1371/journal.pone.0029131 (PMC3253079; doi:10.1371/journal.pone.0029131)
Supplement: Supporting Information S1 — Graphical Abstract. (DOC) [file pone.0029131.s001.doc]

**GRAPHICAL ABSTRACT**

**Using hydrophilic ionic liquid, [bmim]BF_4_ – ethylene glycol system as a novel media for the rapid synthesis of copper nanoparticles.**

Manika Dewan, Ajeet Kumar, Amit Saxena, Arnab De,^#^ Subho Mozumdar[[1]](#footnote-2)*

Department of Chemistry, University of Delhi, Delhi, India

^#^Department of Microbiology and Immunology, Columbia University Medical Centre, New York, USA

E-mail*: [subhoscom@yahoo.co.in](mailto:subhoscom@yahoo.co.in)

Hydrazine

CuSO_4_.5H_2_O

Cu (0) nanoparticles

+ ionic liquid

1. ^*^ [↑](#footnote-ref-2)
